# The role of the complement system in Multiple Sclerosis: A review

**DOI:** 10.3389/fimmu.2022.970486

**Published:** 2022-08-10

**Authors:** Nil Saez-Calveras, Olaf Stuve

**Affiliations:** ^1^ Department of Neurology, University of Texas Southwestern Medical Center, Dallas, TX, United States; ^2^ Neurology Section, Veterans Affairs (VA) North Texas Health Care System, Dallas, TX, United States

**Keywords:** complement system, multiple sclerosis, progressive multiple sclerosis, EAE (experimental autoimmune encephalomyelitis), synaptic pruning, epstein barr virus, complement inhibition, immunosenescence

## Abstract

The complement system has been involved in the pathogenesis of multiple neuroinflammatory and neurodegenerative conditions. In this review, we evaluated the possible role of complement activation in multiple sclerosis (MS) with a focus in progressive MS, where the disease pathogenesis remains to be fully elucidated and treatment options are limited. The evidence for the involvement of the complement system in the white matter plaques and gray matter lesions of MS stems from immunohistochemical analysis of post-mortem MS brains, *in vivo* serum and cerebrospinal fluid biomarker studies, and animal models of Experimental Autoimmune Encephalomyelitis (EAE). Complement knock-out studies in these animal models have revealed that this system may have a “double-edge sword” effect in MS. On the one hand, complement proteins may aid in promoting the clearance of myelin degradation products and other debris through myeloid cell-mediated phagocytosis. On the other, its aberrant activation may lead to demyelination at the rim of progressive MS white matter lesions as well as synapse loss in the gray matter. The complement system may also interact with known risk factors of MS, including as Epstein Barr Virus (EBV) infection, and perpetuate the activation of CNS self-reactive B cell populations. With the mounting evidence for the involvement of complement in MS, the development of complement modulating therapies for this condition is appealing. Herein, we also reviewed the pharmacological complement inhibitors that have been tested in MS animal models as well as in clinical trials for other neurologic diseases. The potential use of these agents, such as the C5-binding antibody eculizumab in MS will require a detailed understanding of the role of the different complement effectors in this disease and the development of better CNS delivery strategies for these compounds.

## Introduction

Multiple sclerosis (MS) is a chronic inflammatory disease characterized by the autoimmune destruction of myelinated axons in the Central Nervous System (CNS). Its course is highly unpredictable, but most patients initially present with episodes of reversible neurological deficits followed by a progressive stage. The role of the complement system in the pathogenesis of MS is complex. Its dysregulation in the gray and white matter of MS patients may play a role in the different stages of the disease. It may also be a contributing component in progressive MS, where the disease process is less well understood and effective treatments are lacking. In this review, we aim to characterize the potential involvement of the complement system in MS with a focus in the progressive forms of the disease. We begin by defining the current understanding of the pathogenesis of progressive MS and how myeloid senescence may be shaping this condition. We then review the existing evidence for the role of complement in MS, its interaction with senescent microglia, and the possible sources of these complement proteins in the MS brain. We also examine the intersection between the complement system and Epstein-Barr virus (EBV) infection, a well-recognized risk factor for MS. Finally, we discuss the complement inhibitor therapies that have been tested in Experimental autoimmune encephalomyelitis (EAE) animal models and in clinical trials for other neurologic disorders with the aim of determining whether its use could be broadened to the management of patients with MS.

## Progressive multiple sclerosis and myeloid senescence

Most patients with multiple sclerosis (MS) present with relapsing-remitting MS (RRMS) on their initial visit. However, with time, the disease eventually progresses to a secondary progressive form (SPMS), with some individuals developing primary progressive multiple sclerosis from the onset (PPMS). These patients lack the clinical and paraclinical magnetic resonance imaging (MRI) evidence of disease activity. Over the past two decades, the advent of disease modifying therapies (DMT) has succeeded in controlling RRMS. However, these therapies have performed poorly in preventing disease progression and clinical disability in the progressive forms of MS. This suggests that different disease mechanisms may be ongoing. As reviewed by Morgan et al. (1), one possibility is that the inflammatory targets that were relevant in RRMS are no longer active. Another is that the same inflammatory response may still be involved but these processes become increasingly compartmentalized in the CNS behind a relatively preserved Blood Brain Barrier (BBB). Autopsy studies from progressive MS patients have revealed that the focal white matter plaques characteristic of RRMS no longer have a prominent inflammatory component in progressive MS but rather experience a slow and gradual expansion at their margins. These lesions have their border lined by chronically activated microglia and myeloid cells (2, 3) with a phenotype resembling that seen in neurodegenerative disease (4, 5). This senescent or “aged” microglia (5) have a secretory phenotype characterized by increased production of pro-inflammatory cytokines and reactive oxygen species (ROS), elevated iron storage and decreased effective phagocytic activity and motility. This decreased ability to effectively clear myelin degradation products by microglia may compromise remyelination efforts in progressive MS (6–8). This phenotype may however be reversible; *in vitro* models revealed that incubation of senescent aged microglia with young microglia medium rescued the ability of old microglia to clear amyloid-β (Aβ) (9). Failure of remyelination may also be secondary to the conversion of oligodendrocyte progenitor cells (OPCs) and neural progenitor cells (NPCs) to a senescent state that prevents OPCs from transitioning to a myelinating phenotype (10). Interestingly, these cells can also induce the senescence of neighboring cells through a paracrine mechanism (11). Another characteristic feature of progressive MS is the presence of inflammation accumulating throughout the whole brain and associated with slowly progressive axonal injury. This eventually leads to diffuse atrophy and demyelination of the gray matter and the so-called normal appearing white matter (NAWM). Cortical demyelination can affect both the forebrain and cerebellum and involve up to 60-90% of the total cortical area in progressive MS (2). These cortical lesions can appear as contiguous lesions with the subcortical white matter, perivascular small lesions confined to the gray matter, or as lesions extending from the pial surface to the deeper cortical layers in the cortical sulci. They are characterized by the presence of profound microglial activation in close proximity to neuronal dendrites with scarce T and B cells (12, 13). NAWM is affected by generalized inflammation and microglial activation causing diffuse axonal injury and destruction, followed by secondary demyelination (14, 15). Inflammatory infiltrates consisting of CD8+ T lymphocytes and activated microglia clusters and nodules are usually observed. In NAWM, microglia-induced radical-mediated mitochondrial injury may be the driving factor leading to axonal dysfunction (16, 17). Overall, microglia in the progressive MS brain appears to mediate a chronic inflammatory state characterized by loss of immune and neuronal function, failure of remyelination, impaired BBB integrity and ultimately neurodegeneration resembling the phenotype seen in neurodegenerative disease (18). As it has been extensively described in Alzheimer’s disease (AD) and other neurodegenerative conditions (19, 20), the complement system may be playing a critical role in the modulation and effect of this microglia phenotype in progressive MS lesions.

## Complement in the white matter of MS: A focus in progressive MS

The role of the complement system in MS was characterized by Lucchinetti et al. who revealed that MS brains had complement deposition in the pattern II subtype of demyelinating white matter plaques (21). More recently, consistent complement and Ig deposition was also described across all areas of demyelination regardless of the plaque subtype, thus implying that the complement system may be involved in MS once the disease is established (22). In this study, complement and antibodies colocalized with phagocytic macrophages/microglia suggesting that complement-mediated myelin phagocytosis may be a critical mechanism for demyelination in MS (22). Evaluation of secondary progressive MS cases also revealed the presence of C3d in periplaque white matter areas with ongoing demyelination (23). Co-localization of IgG and complement was inconsistent suggesting that C3d may act as an opsonin against myelin antigens independently of antibody binding (23, 24). In line with this, Ingram et al. revealed that, in patients with progressive MS and longstanding disease, white matter plaques were consistently positive for complement proteins (C3, Factor B, C1q), activation products (C3b, iC3b, C4d, TCC) and regulators (factor H, C1inh, Clusterin) (25). These markers were positive in chronic active and inactive plaques and NAWM despite the absence of other inflammatory components such as lymphocytes, plasma cells or foamy macrophages. This provided evidence that, once established, progression of inflammation in MS may not rely on infiltrating cells but rather on innate immune mechanisms including complement activation (25). Most of the complement immunolabelling in MS was cell-associated either on the surface or within the cell. Remarkably, complement stained (C1q, C3, iC3b, TCC) reactive GFAP+ astrocytes were found in the white matter plaques of MS patients (25). These complement-positive reactive astrocytes, also named A1 astrocytes, are neurotoxic and were often found near clusters of HLA+ activated microglia and macrophages containing complement-stained debris. This positive staining of reactive astrocytes for complement activation products seems specific to demyelinating disease as it was not observed in control patients or in other neuroinflammatory diseases such as AD, ischemia or encephalitis. Using Tissue MicroArray and IHC, C1q and C3b/iC3b were also found to colocalize with reactive astrocytes, as well as microglia and neurons (26). Depletion of astrocytes ameliorates the disease phenotype in experimental autoimmune encephalomyelitis (EAE) models of MS (27, 28). This suggests a potential crosstalk between astrocytes and innate immune mechanisms in MS, whereby microglia and macrophages induce a chronic pro-inflammatory environment which leads to pathogenic astrocyte activation. These activated astrocytes in turn allow the entry of more pro-inflammatory monocytes to the CNS, serve as Antigen Presenting Cells (APC) through MHC-II expression (29) and acquire a secretory phenotype (6, 7) with production of inflammatory molecules. Evidence for a critical role of the complement system in this cross-talk between microglia and astrocytes has already been described in AD (30) and could be relevant in progressive MS as well. These astrocytes can also downregulate glutamate and potassium transporter expression in the cell surface leading to excitotoxicity (31). Glutamate excitotoxicity decreases the threshold for complement activation on the surface of neurons (32) and is a recognized mechanism of neurodegeneration in MS (33). Therefore, the pathogenic role of astrocytes in MS could, at least in part, be mediated through the activation of the complement cascade.

Evidence for the involvement of the complement in MS also comes from studies on CSF and blood biomarkers. The levels of plasma and CSF C4a were found to be elevated in the brains of patients with RRMS when compared to controls. This elevation was seen only in the acute relapse phase of the disease, decreasing approximately two to three months after (34). C3 levels were also significantly elevated in the CSF of patients with progressive MS (35) and soluble C5b-C9 terminal complexes were also increased in the CSF of MS patients (36). Factor H levels predicted disease progression (37) as its levels steadily increased over a 2 year period in PPMS and SPMS and were significantly higher in these patients when compared to RRMS (38). Significant increases in sCR2 have also been detected in the CSF of either RRMS or SPMS patients (39).

The role of the terminal complement components appears to be more prominent in the white matter than the gray matter of patients with MS. Interestingly it seems that the neurotoxic effects of the terminal complement (MAC) may be concentration dependent. As described below, C5-deficient mice have greater inflammatory demyelination and axonal loss, with more oligodendrocyte apoptosis, than controls (40). Some studies seem to suggest that this is the result of sub-lytic MAC levels conferring protection against OGD apoptosis (41). These effects are largely mediated by low levels of MAC acting *via* regulation of caspase activity (42), inhibition of cytochrome c release (41) and FasL-TNFα signaling.

The underlying mechanism for complement activation and deposition in white matter lesions remains to be elucidated but the pro-inflammatory state induced by cytokine-secreting senescent myeloid cells at the edge of the lesions may play a role. These microglia have an altered surveillance phenotype with less dendritic branching and mobility, impaired phagocytosis and a sustained inflammatory response in reaction to damage which could potentially lead to aberrant complement proteins’ secretion and activation (43, 44). Others have hypothesized that, rather than an active pathological mechanism, C3d+ microglia clusters found in chronic WM plaques could actually represent a physiological response to remove irreversibly damaged axons (45) given these clusters were found in chronic but not in acute lesions in MS (45). We argue that both hypotheses could be true and occurring simultaneously. It is a possibility that complement activation would be purposely aimed at clearing myelin products and other toxic species. However, the ineffective clearance of these complement-targeted myelin degradation products by senescent microglia would lead to a sustained activation of the complement response eventually leading to terminal complement production, C3a and C5a chemokine synthesis, and axonal degeneration. Blood derived fibrinogen deposits have also been found at the edge of these chronic smoldering lesions (7, 46) and this protein constitutes a potent complement activator (47). These findings also suggest that a constantly leaky blood-brain barrier (BBB) due to accumulation of senescent endothelial cells with an impaired tight junction structure may be present in the brains of progressive MS patients (48). Fibrinogen can also interact with microglia *via* the CD11b/CD18 integrin receptor leading to perivascular microglial activation and axonal loss (49), which can contribute to the maintenance of the chronic inflammatory phenotype of these cells.

## Complement in the gray matter and hippocampus: Synaptic pruning and progressive MS

Gray matter (GM) cortical lesions in progressive MS can have different presentations. Among them, the most intriguing are subpial cortical lesions, which represent 70% of the GM demyelination. Subpial cortical demyelination and degeneration, which is more dominant in the later progressive disease (12, 50), is associated with neuronal (51), axonal (52), and synaptic injury (53, 54) and plays an important role in MS progression (55). One of the areas that is most prominently affected in progressive MS with extensive demyelination is the hippocampus (54, 56). This potentially explains the memory and cognitive impairment that occurs in patients with PPMS and SPMS. It has been suggested that the synaptic abnormalities that occur in the gray matter of these patients can, at least, be in part mediated by the complement system (12, 57).

The role of the complement system in neuronal synapse removal was initially proposed by Stevens et al. (1). In their classical study, C1q and C3 were found to be critical in the synaptic engulfment process, also known as synaptic pruning, that leads to the elimination of inappropriate synaptic connections by complement receptor 3-positive (CR3+) microglia during neurodevelopment. This complement-mediated targeting of synapses for elimination may also be aberrantly activated in neurodegenerative disease (58). This has been extensively described and reviewed in postmortem studies and experimental models of AD (59–61).

Michailidou et al. (62) established that the complement system, and in particular the C1q-C3 axis, may also be activated in the hippocampi of MS patients. C1q and C3d colocalized with synaptophysin-positive synapses, HLA+ microglial processes and lysosomes. The neurons in this area also exhibited signs of damage with reduction in their presynaptic terminal density and increased immunoreactivity for mitochondrial stress protein mtHSP70. These changes were mostly localized to the CA3/2 and CA1 regions, which are the most affected areas in progressive MS. This suggests that in the hippocampi of MS patients, complement may tag synapses for engulfment by microglia. This occurs in a process that is independent of the activation of the terminal MAC (C5b-C9) complex. MS hippocampi with no other concurrent neurological diagnosis were consistently negative for C5b-C9 staining (62). This is consistent with a prior study in which C1q and C3d were detectable, albeit at a low level, in combined gray/white matter plaques, whereas C5b-C9 was always absent from these cortical lesions (63). These findings contrast with what is seen in AD where C5b-C9 deposits colocalize with amyloid plaques, neurons and microglia clusters in the hippocampi leading to inflammatory foci and neuronal loss (64).

In line with the above findings, Watkins et al. described that C1q, Bb and C3b-immunopositive neurons were increased in gray matter lesions of patients with progressive MS (65). HLA-D+ phagocytes were seen in close contact to C3b+ cortical neurons and the density of activated myeloid cells (C5aR+, HLA-D+) correlated with the number of classical and alternative complement positive cells in these lesions. C3b-stained myelin was also present in close apposition with HLA-D+ phagocytes, oligodendrocytes (Olig-1+), and microglia (Iba-1+). Cortical layers V-VI near the white matter border were the most reactive, but this process was independent of the presence of underlying white matter lesions, suggesting that complement-mediated synapse elimination and cortical degeneration may be an independent process from white matter pathology in MS. The number of complement regulator C1-Inh+ and factor H+ neurons was unchanged between MS patients and controls, while the density of the terminal complement regulator Clusterin (Clu) was increased. This suggests that an imbalance between complement activation and regulation may be ongoing in the gray matter of patients with progressive MS. The upregulation of Clu provides evidence for the suppression of the terminal complement in cortical areas (66). However, in contrast to prior studies, C9neo+ neurons were elevated in deep cortical and subpial lesions in comparison to non-inflammatory controls, indicating that at least to some degree, MAC formation may occur in the gray matter. *In situ* hybridization for C1qA mRNA revealed that this protein was synthesized by neurons of the deep cortical laminae. Intracellular C1q can be recognized by the mitochondrial C1q receptor, which can trigger ROS production and mitochondrial DNA damage leading to subsequent neuronal dysfunction or death (67, 68). Thus, apart from the effects of extracellular complement on synapse elimination and neuronal death, intrinsically produced complement may be acting as a death signal to neurons.

The trigger for complement deposition in the neuronal synapses of cortical lesions in progressive MS remains to be elucidated. One hypothesis is that aberrant complement-mediated synapse elimination is mediated by senescent microglia. Microglia has a critical role in the maintenance of synaptic plasticity both during development and adulthood (69). Aged and iron-loaded microglia secrete IL-1β (70) and ROS (71) which have been shown to impair long-term potentiation in the hippocampus. These effects could be due to an aberrant activation of the complement cascade by these senescent microglia leading to inappropriate synaptic elimination. Another hypothesis is that complement activation may be a secondary event initially aimed at attempting to effectively clear compromised neurons. In subpial cortical lesions, the neuronal soma of progressive MS patients demonstrates hallmarks of cellular senescence including the presence of cytoplasmic lipofuscin (72). Complement proteins are specialized in targeting these senescent and apoptotic cells (67). Its dysregulation in the context of chronic inflammation, however, would lead to the cortical degeneration seen in progressive MS.

## Source of complement proteins: Systemic or intrinsic

The source of the complement proteins in MS has been a topic of extensive debate. While it appears that in other CNS conditions, such as Neuromyelitis Optica (NMO), BBB disruption leads to systemic complement protein deposition, this may not be the case for MS. This was extensively reviewed by Morgan et al. (1).

BBB breakdown has been described in RRMS patients using MRI studies with gadolinium (73). During the initial stages of MS, vascular endothelial cells express proinflammatory chemokines (i.e. CCL9, CXCL12, CCL21), that recruit T cells into the CNS. These primed T cells can in turn produce proinflammatory cytokines (i.e. TNF, GM-CSF) that promote the recruitment of myeloid cells from the peripheral blood. This process is in part mediated by the expression of matrix metalloproteases (MMP-2, MMP-9) that selectively disrupt astrocyte foot processes leading to increased BBB permeability. This BBB disruption may potentially lead to the egression of soluble plasma components such as autoantibodies, complement proteins and coagulation factors into the CNS. The presence of a meningeal tertiary lymphoid tissue (TLT) has been identified in MS patients (52). In EAE models, this tissue harbors C3+ cells (74). In addition, meningeal inflammation in MS correlates with cortical neuronal loss following a gradient pattern (51, 75). Proteomic CSF profiling of MS patients revealed that proinflammatory cytokines (CXCL12, TNF, IFN, CXCL12, IL6, IL8, IL10) (75), and signature proteins involved in the complement, coagulation cascade (including fibrinogen) and iron homeostasis were elevated in patients with a high cortical lesion load (76). It was thus hypothesized that soluble factors including complement proteins produced in this TLT could be diffusing from the meninges into the subpial cortex (76). The distribution of cortical lesions could thus be dependent on CSF flow and local stasis of these inflammatory molecules.

**Figure 1:**
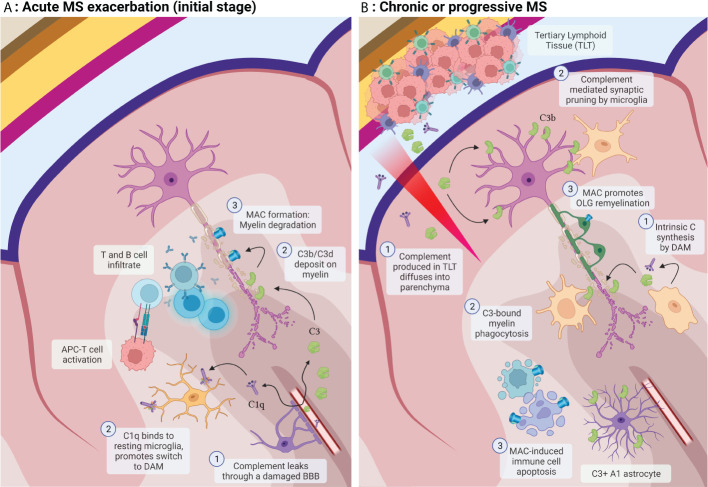
**(A)** Acute MS exacerbation: 1) Complement factors leak through a compromised BBB. T and B cells infiltrate the parenchyma and are activated by myeloid APCs. 2) Activated C3b and C3d deposit on myelin promoting its opsonization. C1q binds resting microglia and modulates its phenotype switch to disease-associated microglia (DAM). 3) Downstream activation of complement leads to MAC formation and damage to the myelin membrane. **(B)** Progressive MS: 1) Complement and other factors are secreted by DAM and by the tertiary lymphoid tissue (TLT) and diffuse into the brain parenchyma. 2) C3-bound myelin products are opsonized by myeloid cells and activated microglia. 3) In this stage, MAC formation exerts protective effects through the apoptosis of inflammatory cells and prevention of OLG apoptosis (Created with BioRender.com).

The patients described in the above study had RRMS. It is thus questionable whether the same mechanisms lead to cortical damage in progressive MS. In these patients, the source of these complement factors could otherwise be intrinsic. Although serum-derived complement can leak through a compromised BBB caused by senescent endothelial cells (48) and astrocytes, or migrate from the TLT through a highly permeable meningeal brain barrier, parenchymal cells can also produce these complement components in the brain. Astrocytes can secrete C3 and factor B (77), microglia express C1q, C3 and C4 (78) and both cells contain receptors for complement activation products (C3dR, C3aR, C5aR). Neurons can also express complement products and regulators (C1q, C3, C4, C5, C6, C7, C9, factor B, factor H, C1inh) (79), and oligodendrocytes can produce membrane complement regulators CD59, DAF, membrane cofactor protein (MCP), C1inh, Vitronectin and Clusterin (80). Complement expression in these cells can be upregulated in diseases such as AD or after pro-inflammatory cytokine stimulation (81). As mentioned above, C1q synthesized by neurons can also serve as an autologous apoptotic signal through its binding to C1qR in mitochondria. Therefore, in patients with progressive MS, the complement cascade may be intrinsically activated by degenerating neurons and exposed myelin without the need for peripherally derived autoantibodies or other factors (23, 24). **Figure 1** reviews the role of complement in acute and chronic MS.

## EBV, HHV-6 and MS: further suggestion of a potential critical complement role

Complement-bound products cannot only be recognized by phagocytes but also by T and B cells through their complement receptors. The CR2 receptor (CD21) on the surface of B cells serves as the link between the complement-mediated immune response to pathogens or other foreign antigens and the adaptive immune response. Binding of complement fragments C3d, iC3b and C3dg to CR2 (CD21) can lower the threshold for B cell activation (82) and complement products can also modulate T cell activation (83). Several infectious agents including the Epstein Barr Virus (EBV), Human Immunodeficiency Virus (HIV) and prions can also bind to the complement receptor CR2 in these cells either directly, through EBV gp350/220 in case of EBV, or indirectly by means of complement-bound immune complexes (84). EBV has emerged as a potential critical risk factor for MS. The risk of developing MS can be increased by up to 32-fold after infection with EBV, but not after infection with other viruses such as CMV (85–87). Interestingly, MS risk is lower among CMV-positive than among CMV-negative individuals (88). Studies suggest that EBV flares may also correlate with MS relapses (89) and the detection of anti-EBV antibodies in peripheral blood correlates with the onset of MS (90). These antibodies can also be found in the CSF of these patients (91). In mice models, transfection of humanized EBV+ peripheral bone marrow cells to EAE mice resulted in a worse disease phenotype than in those mice transfected with EBV- cells (92) (*preprint*). This evidence points towards an important role of EBV in MS pathogenesis. There is also substantial evidence that infection with EBV is linked to a high risk of other autoimmune conditions including SLE, RA or Sjögren’s disease (93).

One hypothesis behind these findings is that, as it is the case with the binding of complement proteins to CR2 in B cells, binding of EBV to this receptor during B cell infection can potentially lower the threshold for the activation of these cells. EBV binding to B cells through CR2 can activate NF-kB and other proinflammatory signaling pathways that lead to the release of proinflammatory cytokines including IL-6, TNFα or GM-CSF (94). It is plausible that the virus could infect a senescent B cell pool reactive against a CNS self-antigen target. This would lead to the selective proliferation of this population and the subsequent development of CNS autoimmunity.

EBV-infected B cells are targeted and eliminated by CD8+ cytotoxic T cells. Another suggestive hypothesis is that the chronic infection of these activated autoreactive B cells can eventually lead to T cell killer response exhaustion. These B cells would then no longer be eliminated and accumulate in lymphoid tissues and in the MS brain (93, 95). A clinical trial of autologous EBV-specific T cell therapy against infected B cells is currently ongoing (96).

Once the inflammatory response is underway and complement activation occurs, fragments iC3b, C3d, C3dg could be self-perpetuating the activation of these auto-reactive B cells through CR2 binding independently of EBV activity. CR2-bearing T cells could also be activated, thus contributing to the pathogenesis of autoimmunity (97, 98). Therefore, specifically targeting complement binding to CR2 may serve as a therapeutic option for MS. It has been proven that the use of conjugated peptides consisting of CR2 bound to complement inhibitors Factor H and Crry rescues the disease phenotype in EAE mice models (99). Another possibility is the use of a decoy soluble CR2 (sCR2). *In vitro*, sCR2 inhibits the cleavage of C3b to iC3b and exerts a modulatory role in complement activation downstream of C3. Its levels are significantly increased in the CSF of patients with RRMS or SPMS and correlate with C1q and C3 levels, and disease severity. Therefore, the use of this soluble receptor as a treatment could modulate MS in two fronts, one by diverting EBV binding from B cells and second by modulating the complement activation cascade (39).

Other theories for the potential role of EBV in MS pathogenesis were extensively reviewed by Bar-Or et al. (89). The molecular mimicry theory presumes that T cells primed by exposure to EBV antigens cross-react to recognize CNS self-antigens. EBV infection may also trigger the expression of self-antigens in B cells, such as αB-crystallin that can be recognized by Th cells and trigger a response against oligodendrocytes, which also express this peptide (100). EBV can translocate to the CNS and TLT within the meninges. The antiviral response against infected cells there could also generate an inflammatory milieu, including complement protein synthesis and secretion, that promotes “bystander” neuroinflammation within the CNS (94).

Another herpesvirus, Human Herpesvirus 6 (HHV-6) is widely spread in the population and has also been associated with neuroimmunologic disorders, including MS (101). Animal and *in vitro* models have demonstrated that CD46, a complement regulator involved in complement inhibition, is a receptor for the HHV-6 (102, 103). CD46 is a membrane-bound complement regulator which serves as a cofactor in the factor I-mediated cleavage and inactivation of C3b and C4b (104). sCD46 and HHV-6 DNA from MS sera were found to co-purify in an immunoaffinity column comprised of immobilized monoclonal antibodies to CD46. This suggested that HHV-6 tightly binds CD46 in the serum of MS patients (105). Expression of CD46 has also been demonstrated in CNS-resident cells. Cultured astrocytes were found to have a strong positive staining for CD46 in IF studies (106). Both variants of HHV-6, HHV-6A and HHV-6B, have a special tropism for astrocytes and OLG in the CNS (107, 108). These cells can act as a reservoir for this virus and both cell types have been found to contain HHV-6 in MS lesions. The pattern of infection of astrocytes is different between the two variants, while HHV-6B causes a nonproductive infection, HHV-6A appears more lytic and leads to a productive infection (109). These different patterns could theoretically lead to different disease patterns and outcomes. It has been described that upon binding to CD46, HHV-6A, causes a rapid downregulation of this receptor that is slower upon infection by HHV-6B (102). Therefore, one may hypothesize that by downregulating CD46 on the surface of astrocytes, HHV-6 infection, and especially HHV-6A, may lead to a dysregulation of complement inhibition in the parenchyma and thus contribute to the inflammatory response seen in MS. Interestingly, astrocytes’ infection by cytomegalovirus upregulates CD46 surface expression (106). Infection with this virus, in contrast to EBV and HHV-6, also appears to be associated with a decreased risk of developing MS (88). Therefore, the differential regulation of CD46 surface expression could potentially underlie one explanation for the different MS susceptibility between these herpesviruses’ infections.

CD46 also appears to have a major role in adaptive immunity. In T cells, CD46 has been proven to be a mediator of the switch of differentiated Th1 IFNγ+ cells to a Tr1 IL10+, regulatory phenotype (110). Interestingly, upon incubation with CD46, Th1 cells from MS patients were found to have impaired IL-10 switching and secretion capability. In these cells, upon CD46 stimulation not only IL-10 secretion but also IL-10R signaling itself was altered (111). Indeed, when Th1 cells were co-stimulated with anti-CD3 and anti-CD46 antibodies, IL-1β and IL-17 expression was induced, promoting a switch to a Th17 phenotype (112). This effect was not observed in healthy T cells. It also appears that surface expression of CD46 may aid in maintaining general immune homeostasis and serve as a stop signal for T cell activation (113, 114). Therefore, the dysregulation of this surface complement regulator by HHV-6 infection may also be contributing to the abnormal T cell responses seen in MS patients.

## Complement inhibition in animal models of neurodegeneration and EAE

Complement system dysregulation is involved in multiple neurodegenerative and neuroinflammatory conditions, as extensively reviewed by Gomez-Arboledas et al. (115). Beyond Anti-AQP4+ NMOSD and generalized myasthenia gravis, where the C5-inhibitor antibody eculizumab has been FDA-approved for treatment, inhibition of the complement system has also been explored in multiple other neurodegenerative and neuroinflammation models, including AD and MS. **Table 1** provides a summarized comparison of the different effects of complement factors’ inhibition in AD and MS animal models. In AD P301S and APP/PS1 mouse models, C3KO resulted in synaptic preservation, maintenance of hippocampal tissue area and rescue of neurocognitive deficits (116–118). In contrast, in other studies C3-/- APP mice exhibited and increase in total and fibrillar Aβ, Iba1+ microglia and GFAP+ astrocytes (119), while the total number of neurons as assessed by NeuN and MAP-2 were decreased in hAPP mice treated with the C3 inhibitor sCrry (120). C1QKO APP mice models exhibited decreased CD68+ microglia colocalizing with synapses, and decreased synapse loss (20). Fonseca et al. found that C1qKO APP mice harbored similar levels of fibrillary Aβ but had a lower decrease in synaptic markers in the CA3 hippocampus as well as lower activated glia surrounding the plaques (121). The use of C1q blocking antibody was able to inhibit microglial synapse removal in cultured neurons and in Tau-P301S mice, thus preserving synapse density (122). However, other models suggested that C1q may actually decrease fAβ and oligo Aβ neurotoxicity by preventing the interaction between toxic Aβ species and neurons and promoting Aβ aggregation outside of the cell (123). Tenner et al. in their review (60) proposed that this double-edged sword role of the complement system in AD may be explained by the fact that this system could have different stages as the disease progresses. Initially, the upstream complement effectors C1q and C3 may have neuroprotective properties by promoting the engulfment and phagocytosis of pathologic fAβ and Tau species, neuronal apoptotic products and vesicular blebs. Thus, these proteins may serve as scavengers cleaning the brain parenchyma from inflammatory products. Factor H, C1inh and C4BP have been found around plaques in this disease which suggests a potential role of complement regulators in modulating the activation of the complement pathways (124). As the disease progresses these factors may be eventually consumed, leading to the downstream activation of the complement cascade. The downstream products of the complement cascade may promote pro-inflammatory mechanisms leading to neurotoxic effects. These pro-inflammatory effects may be largely mediated by the formation of the MAC on the surface of neurons and by C5a binding to C5aR1, which has been shown to promote pro-inflammatory cytokine secretion by microglia and neuronal dysfunction (125). C5arR1KO in the Arctic APP mice models of AD preserved neuronal complexity and rescued cognitive performance (125). In the Tg2576 mice model the use of the soluble C5aR1 inhibitor PMX205 decreased fibrillar amyloid, activated microglia and astrocytes and prevented cognitive loss (126). Similarly, deletion of C3aR the receptor for C3a, the other classic complement chemokine, also resulted in attenuation of tau pathology, rescue of neuronal deficits and glial reactivity in PS19 AD mice (127).

**Table 1:** Descriptive comparison of the effects of complement inhibition in AD and MS animal models.

ALZHEIMER’S DISEASE MODELS				MULTIPLE SCLEROSIS MODELS		
Inhibition	Mice model	Effect		Inhibition	Animal model	Effect
C1q -/-	APP	Decreased phagocytic microglia and synapse loss. Prevention of synaptic Aβ oligomer toxicity, increased hippocampal LTP ^(20)^		C1q -/-	MOG_35-55_ induced EAE mice	Density of Iba1+ cells, microglia with reactive gliosis morphologies, expression of DAM marker CLEC7A lower in C1q -/- mice. No effect on disease phenotype ^(138)^
	APP APP/PS1	Comparable total and fibrillary Aβ, lower level of periplaque activated glia. Lower decline in synaptophysin and MAP2 in hippocampus CA3 ^(119)^				
	3xTgBUB (APP Swedish, P301L Tau, PSN1 mutation)	C1q addition in cultures protected neurons against fibrillary and oligomeric Aβ toxicity. Enhanced Aβ aggregation outside the cell. Effect mediated by LRP1B, GPR6 ^(121)^				
C1 blocking antibody	Tau P301S	Inhibition of microglial engulfment of synapses and prevention of decline in synapse density ^(120)^		ANX-M1.21 (C1q blocking antibody)	MOG_35-55_ induced EAE mice	Decreased Iba1+ and Iba1+/FTL+ microglia ^(138)^
	
C3 -/-	Tau P301S	Decreased neuron loss, brain atrophy, improved neurophysiological and behavioral measurements ^(114)^		CVF (depletes C3)	Myelin + CFA immunized Lewis rats	CVF given at day 9 delayed onset of EAN by 2-3 days, when given at days 9-12 delayed onset by 4-5 days ^(132)^
	PS2/APP	Rescued plaque associated synapse loss ^(114)^			BPN myelin immunized rats	Lower clinical scores, less demyelination. Fewer ED1-positive macrophages, CD11bc-positive cells ^(133)^
	C57BL/6J	Absence of age-dependent synapse and neuron loss in hippocampal CA3; significantly enhanced LTP and cognition, less anxiety ^(215)^		C3-/-	MOG_35-55_ induced EAE mice	In both C3 -/- and factor B -/- mice, little infiltration of the parenchyma by macrophages and T cells, protection from demyelination ^(135)^
	hAPP	Decreased phagocytic microglia, decreased early synapse loss ^(20)^				Mice equally susceptible to EAE. No differences in production of proinflammatory cytokines (IL-2, IL-4, IL-12, TNF-a, and IFN-y) ^(136)^
		Increased total and fibrillary Aβ plaque burden, insoluble Aβ42, plasma Aβ, loss of neuronal-specific NPP+ neurons in hippocampus, activation of microglia to alternative phenotype (CD45+, decreased CD68) ^(117)^				
sCrry (C3 inhibitor)	hAPP	Increased Ab deposition (2-3x), accumulation of degenerating neurons ^(118)^		CR2-Crry	MOG_35-55_ induced EAE mice	Synaptic preservation in LGN where CR2-Crry AAV injected. Reduced synaptic terminal engulfment within microglial lysosomes. Visual acuity preservation. No effect on demyelination, axonal loss, gliosis, myelin engulfment ^(57)^
						Administration prior to and during onset of EAE attenuates both MOG-induced and transferred EAE in CR2-Crry and CR2-factor H treated mice ^(99)^
						
C3aR-/-	Tau PS19	Tau pathology rescue, amelioration of synaptic impairment and neuronal loss. Reversed disease-associated microglia phenotype and A1 astrocytosis. Effects mediated by signaling pathway involving STAT3 ^(125)^		C3aR -/- C3a CNS expression	MOG_35-55_ induced EAE mice	C3aR -/- attenuated chronic EAE, modestly reduced macrophage and T cell infiltrates in the SC. Selective C3a-GFAP expression exacerbated chronic EAE, mortality, increased macrophage and T cell infiltrates ^(172)^
				Dual C3aR -/- C5aR -/-	MOG_35-55_ induced EAE mice	Delayed onset of disease but no attenuation of disease severity. Greater infiltration of CD4+ T cells ^(173)^
						
C5aR -/-	Arctic APP	Prevention of behavioral deficits. Absent CCR2+ monocytes/macrophages near plaques. Rescue of neuronal complexity. Decreased inflammatory microglia ^(123)^		C5aR -/-	MOG_35-55_ induced EAE mice	Mice fully susceptible to MOG-induced EAE, no difference in disease onset or severity. Similar macrophage and T cell infiltrates. Equal proinflammatory gene expression ^(171)^
				C5 -/-	Guinea pig myelin + incomplete Freund’s adjuvant immunized mice	*Acute EAE:* Delay in inflammatory cell infiltrates and tissue damage *Chronic EAE:* Axonal depletion and severe gliosis in C5 -/-. Extensive remyelination in C5-sufficient mice ^(146)^
					Myelin-induced EAE mice	Increased TUNEL + apoptotic cells in C5 -/- mice during clinical recovery (lymphocytes, monocytes, OLG) ^(147)^
PMX205 (C5aR1 inhibitor)	Tg2576 3xTg	Reduction of fibrillar amyloid deposits, activated glia. In Tg2576 mice, improvement in behavioral tasks with reduction in pathology. In 3xTg, inhibition also reduced hyperphosphorylated tau ^(124)^		PMX205 (C5aR1 inhibitor)	Biozzi AB/H mice (syngeneic Biozzi AB/H spinal cord homogenate + CFA)	Amelioration of progressive neurological disability (not complete rescue). Reduction of NLPR3 inflammasome, upregulation of PPAR ^(143)^
				AcF-[OPdChaWR] (C5aR inhibitor)	*EAE:* gpBMP + CFA immunized rats *ADEAE:* Additional injection of Z12 (anti-myelin) mAb	Neutrophil response to C5a blocked. No effect on clinical disease or pathology ^(170)^

Green coloring represents a beneficial effect of complement inhibition, while orange coloring implies an overall detrimental or null effect of inhibition. Although most studies appear to suggest a beneficial role of C1q and C3 inhibition in AD and MS models, others imply that these factors may exert some protective functions in these conditions. While complement chemokine inhibition (C3a, C5a) was largely protective in AD, the beneficial effects of this inhibition in MS models have been more controversial.

The benefits of targeted complement deficiency or inhibition have also been proven in other inflammatory events of the brain including stroke (128), brain radiation-induced injury (129, 130) and traumatic brain injury (TBI). In TBI mice models targeted inhibition of MAC formation with chimeric molecules or antisense oligonucleotides, resulted in neuroprotective effects (131, 132). C3 or C3aR deficient mice who were infected with West Nile Virus (WNV) were also protected from WNV-induced synaptic terminal loss (133). The use of complement inhibitors has also been explored in MS models.

### C1 and C3 inhibition in EAE

The first studies that evaluated complement inhibition in MS used Lewis rats immunized with myelin and complete Freund’s adjuvant (CFA) (see **Table 1**). In these models, treatment with cobra venom factor (CVF), which depletes complement protein C3, delayed the onset of experimental allergic neuritis (EAN), decreased demyelination, and reduced phagocytic macrophages and CD11b/CD11c positive cells (134, 135). The soluble recombinant form of the human complement receptor (sCR1) was able to inhibit the deposition of C1, C3 and C9, inhibit CNS inflammation, almost completely block demyelination and reduce the severity of clinical disease in mice models of Ab-mediated EAE (136). Similarly, Nataf et al. demonstrated that C3 -/- and factor B -/- MOG_35-55_-induced EAE C57BL/6 mice had little parenchymal macrophage and T cell infiltration and were protected from demyelination (137). In contrast, MOG_35-55_-immunized C3-/- B6×129/F_1_ mice were equally susceptible to induction of EAE as C3 +/+ mice and had no differences in the levels of proinflammatory cytokines (IL-2, IL-4, IL-12, TNF-a, IFN-y) (138). Further investigation is needed to elucidate whether the complement system is important in MOG-induced EAE mice models. It cannot be ruled out that the absence of effect of C3 -/- in the latter study was actually due to a limitation of this particular mice model in mimicking complement effects in MS (139). Another interesting possibility, is that the upstream complement components may have a double-edged sword effect in this disease similarly to what has been observed in AD. On the one hand, C3 may exert neuroprotective actions through the removal of apoptotic debris and myelin degradation products by microglia. On the other, chronic activation and dysregulation may promote an aberrant response with inappropriate intact neuronal, axonal and synaptic targeting.

The above studies explored the effect of the systemic inhibition of the complement system in MS. Therefore, it also cannot be ruled out that the effect of complement inhibition in EAE models was solely due to an effect on the peripheral immune cell response and disease induction rather than an effect on the brain parenchyma. One option to evaluate this is to use adoptive-transfer EAE mice. These mice are transfected with encephalitogenic CD4+ T cells from a different EAE donor mice and thus systemic complement inhibition or KO would not influence disease induction. In addition, the effects of complement suppression on these mice would be solely dependent on interference with the CNS response rather than on a systemic effect. Another option is to selectively inhibit the complement in the CNS. Wernerburg et al. proved that intraparenchymal inhibition of early complement component C3 in MOG_35-55_-induced EAE mice models prevented synapse loss. In these mice, injection of an adenovirus vector (AAV) containing the C3 inhibitor Crry fused to a CR2 domain which binds activated C3 in the lateral geniculate nucleus (LGN) resulted in selective synaptic preservation in this area. Crry was enriched in the presynaptic terminals where there was also a decrease in C3 deposition. AAV-Crry treated mice also had a reduction in VGlutT2+ and VGlutT1+ synaptic terminal engulfment within microglial lysosomes. This indicates that these mice were protected from complement-mediated synaptic pruning by microglia. This treatment also blocked loss of visual acuity at both the onset and peak of EAE. Interestingly, demyelination, myelin engulfment by microglia, axon loss, gliosis and peripheral immune cell infiltration were unaffected, suggesting that synapse loss is an independent process from demyelination (57). This study also implied that synapse loss in demyelinating disease could be independent of C1q. Only C3 but not C1q colocalized with presynaptic terminal markers and was enriched in synaptic compartments, suggesting that the alternative cascade may be mediating synapse pruning in this disease. These findings resemble what has been described in stroke (128) and TBI models (132). As aforementioned, systemic administration of CR2-Crry and CR2-factor H, which effectively blocked the alternative pathway prior to and during EAE, was also capable of delaying the onset and halting the progression of chronic disease in MOG_35-55_ C57BL/6 mice (99).

This evidence seems to suggest that the targeted inhibition of early complement component C3 in the CNS may help prevent synaptic loss in MS patients. As mentioned, aberrant synaptic pruning in the hippocampus may be the underlying neuropathologic substrate for the cognitive impairment that occurs in progressive MS (62). Therefore, the specific targeting of C3 may be an option for therapeutic interventions in this patient population.

The above studies suggest the importance of C3 and the alternative complement pathway in mediating demyelination and synaptic pruning in EAE. However, C1q and the classical complement cascade may also be exerting relevant functions in these models. C1q may be act as a mediator of microglia activation, and, in EAE, it can modulate their switch to an inflammatory profile resembling that seen in neurodegenerative disease. These cells are also known as disease-associated microglia (DAM) (140). Conditional C1q cKO in mice microglia using the TMEM119-CreER driver eliminated C1q immunoreactivity in the brains, consistent with microglia being the primary source of C1q in the CNS. After EAE induction in these C1qcKO mice, the density of Iba1+ cells and microglia with morphologies consistent with reactive gliosis was significantly decreased (140). The expression of the DAM marker Clec7a was also downregulated. In addition, when a C1q-blocking antibody (ANX-M1.21) was employed in EAE mice one-month post-induction, the number of Iba-1+ and Iba1+/FTL+ reactive microglia were also reduced. Together this data demonstrated a role of C1q in mediating microglia reactivity and inflammation in EAE. However, and interestingly, C1cKO did not correlate with a change in EAE clinical onset or clinical score. Gene expression analysis at 25 days post-EAE induction in mice, when the disease transitions to a chronic phase, has also revealed that complement components C1qa, C1qb, C1qc and C3, chemoattractant cytokines and microglial cells are enriched when compared to sham-immunized controls (141). In a different study, C1qKO did not prevent MOG-induced EAE in C57BL/6 mice. However, infusion of anti-MOG antibodies in these C1q -/- EAE mice did not exacerbate disease severity when compared to WT mice, suggesting that the classical complement system is the dominant effector cascade invoked by demyelinating Abs (142). This evidence seems to suggest that in EAE, C1q is capable of modulating microglial phenotype towards an inflammatory one, but that this effect alone may not be enough to curb disease severity. Moreover, C1q and activation of the classical complement may be the predominant effector pathway of demyelinating antibodies in MS. This is important in MS, where at least 30% of patients have pathogenic immunoglobin G responses, that can cause significant complement-dependent antibody-mediated axonal loss in *in vitro* myelinating cultures (143, 144). These antibodies have been found in acute, chronic active and chronic inactive lesions on axons and oligodendrocytes in demyelinated areas. These immunoglobulin deposits were also present on oligodendrocytes in NAWM in some cases (144). In addition, intrathecal Immunoglobulin G synthesis in patients with newly diagnosed RRMS was associated with a higher risk and shorter time of disability worsening (145). This evidence, along with the proven benefit of B-cell targeted interventions in progressive MS (146), confirms the importance of the humoral response in this disease, of which C1q and the classical complement cascade may be critical effectors.

### Inhibition of the terminal complement components in EAE

Early complement proteins may be involved in synaptic pruning, loss of neuronal connectivity and myelin loss in MS. However, intraparenchymal and systemic inhibition of C3 had conflicting effects on demyelination, axonal loss and peripheral immune cell infiltration in EAE. Conceivably, the downstream effectors of the complement system, including MAC-TCC formation and chemokines C3a and C5a, could be important in modulating these effects in MS and EAE models.

Using Biozzi AB/H mice, Michailidou et al. described that the use of antisense oligonucleotides targeting C6 mRNA outside of the CNS prevented demyelination, axonal damage and synapse loss as assessed by the percentage of SYP reactivity and Iba-1+ microglia activation. C9 reactivity in the CNS was sparse suggesting a drastic reduction of MAC formation. These effects were, at least in part, mediated through the inhibition of the Nod-like receptor protein 3 (NLRP3) inflammasome, which resulted in a decrease of NLPR3 effectors including IL-1β. The anti-inflammatory pathways PPAR and LXR/RXR were also upregulated in these treated mice. It has been hypothesized that the formation of MAC in the cells, with subsequent pore formation, can lead to the release of ATP in the brain parenchyma which can bind the purinoreceptor P2X7 in adjacent glia and activate the NLRP3 inflammasome. Therefore, MAC formation may not only have a deleterious effect through neuronal pore formation but also by promoting a proinflammatory response. Interestingly, the C6 antisense oligonucleotide is not capable of crossing the BBB which implies that these effects were achieved by the inhibition of systemic MAC formation (147).

Following immunization with MBP, C6 deficient PVG/C rats developed significantly milder EAE as assessed by clinical score when compared to C6-sufficient rats (148). These rats exhibited no demyelination, had decreased T cell and macrophage infiltration, and had no C9 detection indicating an inability to form the MAC (149). There was also a reduced expression of P-selectin on the surface of endothelial cells, which suggests that the MAC may also contribute to EAE pathogenesis by promoting proinflammatory cell infiltration to the spinal cord through the expression of adhesion molecules in the endothelium. These rats had similar C3 deposition in the spinal cord, implying that these effects were solely mediated by the downstream effectors of the complement cascade. In line with this, mice deficient in TCC-MAC regulator CD59a, developed a more severe disease with substantial demyelination in acute MOG_35-55_-EAE C57Bl/6J mice, demonstrating that terminal complement system activation may be involved in the pathogenesis of this model (139).

It appears that the terminal complement components may exert its noxious effects during the acute stages of EAE by mediating myelin membrane damage and cell extravasation but may also have a restorative effect during chronic EAE (see **Figure 1**). In a C5-deficient guinea pig myelin-induced EAE mice model, a delay in inflammatory cell infiltration, restricted lesion areas and tissue damage were observed in the acute phase of the disease. However, during the chronic phase, C5-deficient mice exhibited increased inflammatory demyelination and Wallerian degeneration, followed by axonal depletion and severe gliosis, while in C5-suficient mice the acute phase of the disease was followed by axonal sparing and extensive remyelination (150). This effect may be mediated through C5 and TCC-induced apoptosis of inflammatory and degenerating cells (151). Their elimination leads to a more efficient clinical recovery from EAE (152, 153). In myelin induced EAE, C5 deficient and C5 sufficient mice had similar number of total apoptotic cells in the acute stage, while C5 sufficient mice had significantly fewer during recovery. In C5 sufficient mice, Fas positivity was higher than in C5 deficient mice during acute EAE, but this was reversed during recovery. In addition, sublytic levels of C5 and MAC may also protect OLG from apoptosis (40, 154). Significantly fewer TUNEL + OLG, were seen in C5 sufficient when compared C5 deficient during both acute EAE and recovery. In a different study, it C5 promoted axon preservation and new myelin formation, and protected OLGs from apoptosis (155). Sublytic MAC levels may enhance OLG survival by inhibition of the mitochondrial pathway of apoptosis through upregulation of Bcl-2 and inhibition of caspase 3 activation (41, 156), as well as downregulation of Fas-FasL signaling pathways (40). Activation of Fas results in recruitment of FADD to the cytoplasmic tail of Fas (157), which in turns binds procaspase 8 to form the death-inducing signalling complex (DISC) (158). DISC formation leads to caspase-8 activation and induction of apoptosis (159). Regulation of the FADD-caspase 8 proapoptotic signaling pathway is mediated through an intrinsic inhibitor, cellular FLIP (160). It has been found that sublytic C5b-C9 inhibits caspase-8 processing and increases FLIP levels in a PI3K-dependent manner on oligodendrocytes. C5b-C9 is also able to activate the cell cycle in OLG and induce the S phase of the cycle in a c-Jun dependent manner. These pro-survival effects are mediated by the activation of the ERK1 and PI3K/Akt pathways (41, 161, 162). This evidence reinforces the molecular pathways by which sublytic C5b-C9 is capable of inhibiting OLG apoptosis and promote their proliferation, and thus explains its significant role in OLG survival in inflammatory disorders (42). OLG also express high levels of CD55 and CD59 on their surface which makes them resilient to MAC attack.

All this evidence points towards a dual role of MAC in EAE. During the initial stage, this complex exerts deleterious effects through damaging of axonal myelin which, in contrast to OLG, lacks CD55 (163). MAC may also mediate pro-inflammatory effects and immune cell extravasation during the acute phase of the disease. However, as the disease phenotype progresses to a chronic state, this complex can aid in remyelination and axonal preservation efforts.

In order to understand the dual effect of MAC in MS pathogenesis, it is also important to explore its importance in a different demyelinating disease where its role has been well described. This is NMOSD, where the use of the C5-targeted antibody eculizumab is an effective FDA-approved therapeutic option (164). The involvement of the complement system in NMOSD was first described by Lucchinetti et al. with the observation of complement and Ig deposition in a perivascular pattern on the outer rim of hyalinized vessels in the spinal cord of these patients (165). These lesions were positive for the membrane attack complex (MAC) components. This increased reactivity at sites of vessel damage suggested that the perivascular space was the primary site of injury in NMOSD (166). This evidence was later supported by the discovery of the NMO-specific AQP4 IgG antibody, which can fix complement. This antibody targets astrocytes at the BBB of the subarachnoid and Virchow-Robin spaces as well as the extracellular matrix of parenchymal penetrating microvessels in the CNS (167). This can ultimately lead to BBB compromise and leakage of systemic factors including complement into the perivascular parenchyma. Demyelination may occur in NMOSD through a “bystander effect” (168) whereby which complement fixation and activation following AQP4-IgG binding to astrocytes leads to the deposition of MAC in myelin found in close proximity to those targeted astrocytes, resulting in perivascular demyelination. Chemokines C3a and C5a can also recruit activated macrophages, eosinophils and neutrophils to the lesions causing further axonal and oligodendrocyte damage. This pattern of complement deposition and demyelination contrasts with that of MS lesions, where complement is present on degenerating myelin sheaths along the active plaque edge rather than following a perivascular distribution (165). The reason why the spinal cord and optic nerve are preferentially affected in NMOSD may also be secondary to the intrinsic permeability of the BBB at these sites. Those areas lacking an effective BBB, such as the spinal nerve roots, may be more susceptible to have peripheral antibodies and systemic complement gain access and diffuse into the immediate vicinity (169, 170).

The different underlying pathogenesis mechanisms make it difficult to ascertain whether complement therapeutics, such as eculizumab, could be as useful in MS as they have proven to be in NMOSD. NMOSD is characterized by acute relapsing attacks that are primarily antibody-mediated with secondary systemic complement deposition. However, given the described noxious effects of C5 and MAC formation during acute MS and EAE attacks, one can envision a potential benefit of using eculizumab and other C5 inhibitors in this stage of the disease. It, however, remains to be elucidated whether this agent could have negative rather than positive effects on the treatment of progressive MS where MAC may be aiding in apoptotic cell clearance and remyelination efforts.

In addition, and as it will be described in greater detail later on in this review, the presence of underlying blood brain barrier compromise in the brains of patients with NMO is also the likely reason why antibody therapies with complement antibodies (i.e. eculizumab) are effective in this disease. The biggest challenge when developing therapies for central neurological disorders is achieving good CNS delivery. Given that the lesions in NMOSD are primarily perivascular and affect areas of BBB compromise, the use of systemic therapies with antibodies can achieve a selective delivery to those lesions without requiring a good BBB penetration. This represents a hurdle in MS, and especially in progressive MS, where areas of active demyelination are confined behind a relatively intact BBB (171).

### The role of chemokines: C5A and C5AR

C3a and C5a are complement fragments that act as potent mediators of inflammation acting as cell activators and chemokines (172). C5a binds to two receptors on myeloid cells, C5aR1 and C5aR2. C5aR1 (CD88) is a G-protein coupled receptor that can promote a pro-inflammatory response through intracellular Ca^2+^ production, chemotaxis, granule enzyme release and oxidative stress. Otherwise, C5aR2 promotes an anti-inflammatory response through non-G protein dependent signaling that alters proinflammatory cytokine production. Therefore, C5 and C5a inhibition can potentially have differential pro and anti-inflammatory effects at different stages of disease depending on the relative expression of C5a receptors on the surface of immune cells. This makes the pro-inflammatory C5aR1 a more appealing target for directed therapies.

In the study conducted by Michailidou et al. (147) inhibition of C5a receptor 1 with PMX205 was assessed. In these mice, C5aR1 inhibition was associated with amelioration of progressive neurological disability but not complete rescue as it was the case for C6 antisense-treated mice. A reduction of NLPR3 inflammasome gene expression was also present, but there was a higher expression of this pathway when compared to C6 antisense oligonucleotide mice and WT mice. The anti-inflammatory pathway PPAR was also found to be upregulated in these treated mice, but not LXR/RXR as it was the case for C6-antisense mice.

The use of another C5aR1 antagonist, PMX53, was evaluated in rat models of brain inflammation and demyelination. This therapy completely blocked neutrophil response to C5a *in vivo* but had no effect on clinical disease or resultant pathology in either inflammatory or demyelinating rat models (173). In a C5aR KO model of MOG-induced murine EAE, C5aR KO did not influence disease severity, the composition of the inflammatory cell infiltrate or local cytokine production (174). One caveat of this study is the short follow-up post-immunization period (20 days) in these mice, which makes it difficult to assess the long-term effects of C5aR depletion. These findings may also suggest that C5a is not solely required for disease induction or perpetuation in MS and that redundant mechanisms such as C3a signaling may be in place. Deletion of C3aR in EAE mice actually provided some protection from disease as evidenced by an attenuated clinical course and IHC analysis revealing reduced demyelination and infiltration of macrophages and T cells. In addition, selective expression of C3a in the CNS under GFAP also exacerbated disease in these mice who also had higher mortality (175). Given this evidence, Ramos et al. developed a C3aR/C5aR double knockout mice model in which EAE was induced. In these animals, a delayed onset of disease was observed but disease severity was not attenuated (176). One reason behind the relative inefficacy of C3a and C5a targeting in EAE is that these chemokines may have different functions depending on the cell type and stage of disease. For example, it has been described that the transcriptional control of C3aR expression in astrocytes is fundamentally different than that in myeloid cells (177). In spinal cord injury rat models, it was found that C5aR inhibition with PMX205 could have either beneficial or detrimental effects depending on the timing of inhibition, while early C5a blockade resulted in improved recovery, inhibition after 14 days post-injury reduced locomotor recovery and myelination (178). Thus, it can be argued that these seemingly opposed timing-dependent functions of C5a could also be a feature of EAE and MS. Therefore, the potential therapeutic benefit of targeted C5a and C3a inhibition requires a better understanding of the role of these chemokines throughout the disease course in EAE and MS. One possibility to explore this would be to inhibit C5a and C3a at different time points with small molecule inhibitors or conditional KO models and assess its effect on the disease course and demyelination.

## Pharmacological complement inhibitors as potential therapeutic options for MS

The basic research evidence for the use of complement inhibitors in different conditions has translated into clinical practice. Currently, only two complement binding agents are FDA-approved for use. These are eculizumab, an anti-C5 antibody, approved for AQP4+ NMOSD, generalized MG, as well as atypical hemolytic uremic syndrome (aHUS) and paroxysmal nocturnal hemoglobinuria (PNH). The other are the C1 esterase inhibitors (i.e. Cinryze) used in hereditary angioedema. Complement inhibitors are also being evaluated for other neurologic conditions beyond NMOSD and MG, including GBS, ALS and TBI. **Table 2** provides a comprehensive list of all the ongoing and completed trials on the use of complement inhibitors in neurologic conditions. At the present date there has not been any clinical study on their use in MS. The potential benefit of complement inhibitors in CNS disease was also extensively reviewed by Carpanini et al. (179). Interestingly, one of the most well-established compounds for the management of multiple neurological disorders, IVIg, has also been postulated to have effects at the complement system level (180). IVIg is capable of scavenging active complement fragments and anaphylatoxins, as well as regulating C3 activation to prevent immune-mediated damage by opsonization or MAC deposition (181, 182).

**Table 2:** Complement inhibitors in clinical trials for neurologic diseases.

Complement inhibitors in clinical trials for neurologic diseases				
PHASE	DISEASE	INTERVENTION	RESULT/STATUS	CODE
**Phase 2**	NMOSD	Eculizumab	At 12 months, 12/14 of treated patients’ relapse-free, median number of attacks decreased, visual acuity and disability improved	NCT00904826
**Phase 3 (PREVENT)**	NMOSD	Eculizumab	1ary endpoint of adjudicated relapse occurred in 3% (eculizumab) vs. 43% (placebo), time until 1^st^ relapse increased, disability status improved	NCT01892345
**Phase 3 extension**	NMOSD	Eculizumab	96% of patients on eculizumab adjudicated relapse-free at 192 weeks. 95% no disability worsening and greater quality of life (QoL)	NCT02003144
**Phase 2/3**	Pediatric Participants, Relapsing NMOSD	Eculizumab	Recruiting	NCT04155424
**Phase 2/3**	Pediatric Participants, NMOSD	Ravulizumab	Not yet recruiting	NCT05346354
**Phase 3**	NMOSD	Ravulizumab	Active	NCT04201262
**Phase 1**	NMOSD	Cinryze (C1INH) as add-on	Completed, no adverse effects but insufficient efficacy. C1 activity inhibition in serum too low to confer clinical benefit	NCT01759602
**Phase 2**	Generalized myasthenia gravis (MG)	Eculizumab	6/7 patients reached 1ary endpoint of 3-point reduction in Quantitative Myasthenia Gravis (QMG) score, QMG mean change significantly different	NCT00727194
**Phase 3 (REGAIN)**	Generalized MG	Eculizumab	1ary endpoint of MG-ADL mean ranked difference change not met but significant improvement in MG-ADL, QMG, MG-QoL15 sensitivity analysis. 2-3x more patients improved in eculizumab group	NCT01997229
**Phase 3 extension (ECU-MG-302)**	Generalized MG	Eculizumab	MG exacerbation rate reduced by 75%, improvement in ADL, muscle strength, functional ability and QoL. 56% of treated patients achieved minimal manifestations	NCT02301624
**Phase 3**	Pediatric patients, generalized MG	Eculizumab	Active	NCT03759366
**Phase 3**	Generalized MG	Ravulizumab	Active	NCT03920293
**Phase 2**	Generalized MG	ALXN 2050 (Factor D Inh)	Recruiting. Goal >2 MG-ADL score reduction in consecutive 4 weeks	NCT05218096
**Phase 2**	GBS	Eculizumab + IVIg	2/5 treated patients had 1-2 grade improvement on the GBS disability score	NCT02029378
**(ICA-GBS)**				
**Phase 2**	GBS	Eculizumab + IVIG	1ary outcome, ability to walk independently 61% (eculizumab) vs. 45% (control), study did not reach predefined response rate	NCT02493725
**(JET-GBS)**				
**Phase 3**	Severe GBS	Eculizumab	Active. Goal assessment of efficacy and safety with Highest Functional Grade Scale	NCT04752566
**Phase 3**	ALS	Ravulizumab	Terminated (IDMC recommended to discontinue the trial due to ravulizumab lack of efficacy)	NCT04248465
**Phase 2**	ALS	Pegcetacoplan (APL-2), C3 inhibitor	Recruiting	NCT04579666
**Phase 2 (CIAO@TBI)**	Traumatic Brain Injury (TBI)	C1 inhibitor	Recruiting	NCT04489160
**Phase 2**	Multifocal Motor Neuropathy	ARGX-117 (C3 inhibitor)	Recruiting	NCT05225675
**(ARDA)**				
**Phase 4**	Neurologic symptoms in post COVID-19	Ruconest (C1 esterase inhibitor)	Recruiting	N+A3:E26CT04705831

Beyond NMOSD and MG, the use of complement inhibitors has been evaluated in GBS, ALS, TBI, Multifocal Motor Neuropathy and post-COVID19 neurologic symptoms.

**Figure 2:**
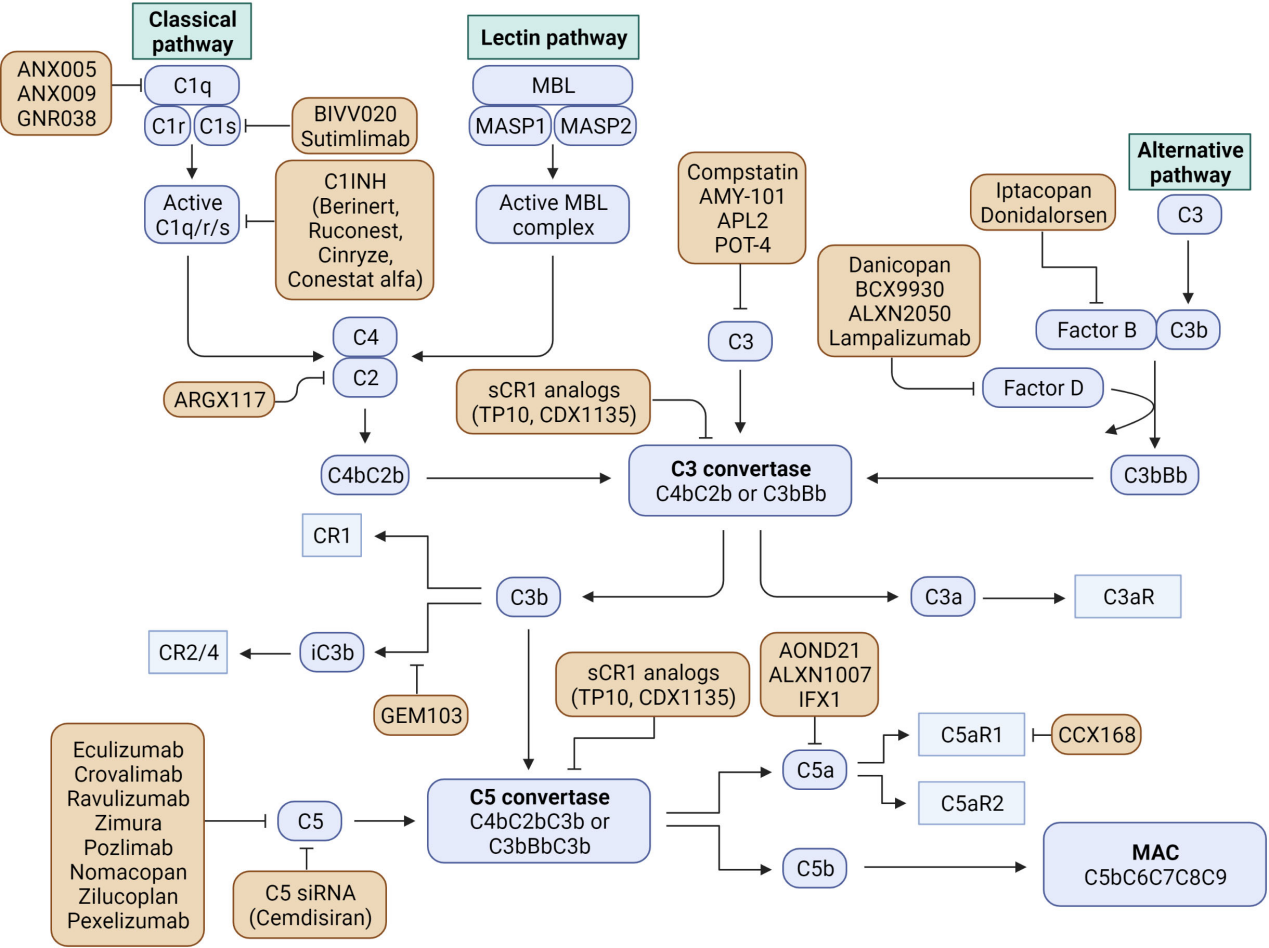
Currently available complement inhibitors used in clinical trials of neurologic and non-neurologic diseases (Created with BioRender.com).

As reviewed by Morgan and Harris (183), the field of complement directed therapies has significantly expanded in recent years. The discovery that age-related macular degeneration can be a target for complement-directed therapies has catalyzed the development of an industry aimed at developing new compounds for this and other chronic conditions. Multiple clinical trials are currently underway with agents directed against different effectors of the complement cascade. Intravitreal injections of the C3 inhibitor Pegcetacoplan (APL-2) and the anti- Factor D antigen binding fragment (Lampalizumab) have shown effectiveness in macular degeneration (184, 185); the C3 inhibitor, APL-2, is also being evaluated for use in PNH and ALS; another C3 inhibitor, AMY-101, has proven effective for the management of gingivitis (186); factor D inhibitor, Danicoplan, has been trialed in PNH (187) and COVID-19, just to name a few. This industry can conceivably serve as a platform for the development of complement-directed therapies for MS that could be used in human clinical trials. **Figure 2** provides a graphical depiction of all the complement-directed therapies on either ongoing or completed clinical trials.

Therapies targeted against systemic complement inhibition carry non-negligible risks including an increased susceptibility to encapsulated bacterial infections, particularly by *Neisseria meningitidis*, as well as immune complex formation and deposition due to impaired clearance (183). One of the possible approaches to prevent these systemic side effects is the direct delivery of drugs to the organ of interest (i.e. the CNS), as it is the case in macular degeneration (184, 185). New therapies have been developed with chimeric agents consisting of a complement receptor ligand bound to a complement inhibitor. This allows for a more targeted approach with increased anti-inflammatory activity without altering the host response to infectious agents (188, 189) A CR2-FH hybrid is currently on clinical trials for PNH (190). As mentioned, the use of these CR2-FH and CR2-Crry conjugated inhibitors has proven to attenuate disease in EAE mice models (99) as well as TBI (132) and SCI (191). Another approach could be the use of complement pro-drugs that are activated on sites of active inflammation (192). Conceivably, these prodrugs could be cleaved into active forms by CNS-resident enzymes such as matrix metalloproteinases (MMP). In MS these enzymes have been found to be upregulated (104). Other approaches include the addition of membrane tags or other modifications to ensure homing of the agent where needed (193).

### Curbing microglia activation with complement inhibitors

As mentioned above, the lesion border of progressive MS lesions is lined by activated “senescent” disease-associated microglia and myeloid cells (2, 3) with decreased phagocytic activity, mobility and branching, and increased production of pro-inflammatory cytokines, ROS and iron storage (4, 5). Transcriptomic studies in MS have shown that these microglia have a partially overlapping phenotype with that of DAM in other neurodegenerative diseases (140, 194). The genes upregulated in some of these microglia populations in chronic active lesions of MS patients included MHC-II class complex proteins, inflammatory markers, factors involved in iron processing and complement C1-complex genes (C1QA, C1QB) (140). These findings were replicated in other studies that revealed enrichment for transcripts encoding activation markers, complement factors, MHC proteins and lipid degradation proteins. Meanwhile, genes involved in synapse remodelling were downregulated (105). In EAE, single-cell RNAseq also showed enrichment of oxidative stress genes, coagulation factors, antigen-presenting and inflammatory markers, as well as decreased expression of homeostatic genes (*P2ry12, Sparc, Cxcr1* and *Tmem119*) (195). This evidence seems to suggest that, in patients with progressive MS, curbing these DAM phenotypes towards a homeostatic profile could serve as an important target for therapeutic interventions. *In vitro* models have already revealed that this phenotype is reversible as evidenced by the ability of young microglia medium to rescue the phagocytic function in senescent aged microglia (9).

Targeting the complement system, and specifically complement C1q, could help modulate these microglia phenotypes. As mentioned above, the use of the C1q-blocking antibody ANX-M1.21 in EAE mice one-month post-induction, led to a reduction in the number of Iba-1+ and Iba1+/FTL+ reactive microglia (140). This identified C1q as a critical mediator of microglia activation in MS and, therefore, a candidate target to curb chronic inflammation in the white matter of this disease. Therefore, the use of C1q inhibitors including C1-INH, already approved for use in PNH, and other small molecule inhibitors (see **Figure 2**) should be also trialed in these mice to determine their ability to switch this DAM phenotype. This effect could have important implications in the management of progressive MS, where this “senescent” microglia appear to have major importance in disease pathogenesis.

Other complement effectors could also be involved in these microglia modulating effects. In AD, deletion of C3aR in PS19 AD mice also resulted in rescue of tau pathology and attenuation of neuroinflammation. RNAseq identified that a C3aR dependent transcription factor network mediated by STAT3 regulates a glial switch which, when inactivate, ameliorates the DAM phenotype and reactive astrocytosis (127). Similar findings were observed when C5aR was KO in these mice, where inflammation related genes were significantly reduced (125). In mice models of spinal cord injury, the use of C5aR1 antagonists PMX53 and PMX205 reduced microglial numbers, the expression of proinflammatory cytokines (IL-1β and TNF) and astrogliosis (196). This evidence seems to suggest that complement chemokine inhibitors could also serve to modulate microglial activation and function (178). However, as discussed above, inhibition of these chemokines in EAE has led to conflicting results, suggesting that these may be exerting complex immunomodulatory functions in this condition, and their inhibition may require a better understanding of disease pathogenesis and timing of intervention. In order to develop these targeted interventions, another of the challenges is to develop EAE models that can reliably mimic the features of progressive MS and the “senescent” microglial phenotypes seen in these patients.

### The BBB challenge: Small peptides, transient opening or trojan horses

Despite the development of new candidate therapies, achieving consistent delivery of these complement inhibitors to the CNS remains the biggest hurdle for neurological diseases. The proven efficacy of eculizumab in AQP4+ NMOSD may be attributed to the fact that there is a significant BBB compromise in this disease perivascular lesions that allows the egression of this complement antibody from the systemic circulation to the areas of active pathology (165, 167, 170). This represents a challenge in MS, and especially in progressive MS, where areas of active demyelination are confined behind a relatively preserved BBB. Therefore, better CNS delivery strategies for targeted therapies need to be developed in this disease.

The BBB precludes the passive entry of molecules larger than 400 kDa. One approach to overcome this is the development of small molecule complement inhibitors that, in contrast to antibodies, could achieve a better delivery to the CNS (197). Compstatin is small cyclic peptide that blocks C3 cleavage (198). This drug and its analogs (POT-4, AMY-101) have been trialed for gingivitis (186), macular degeneration and PNH. Nafamostat mesylate is another small-molecule protease inhibitor trialed for DIC and acute pancreatitis (199). Small molecule inhibitors of factor D are also currently under development (200). Perhaps one of the most well-described small-molecule hydrophobic blockers that have been developed is the cyclic hexapeptide PMX53 that binds and blocks C5aR1 and has good BBB permeability (201). This inhibitor rescued the phenotype of mice models of AD (126), ALS (202), HD (201). As described above, however, this inhibitor had no effect in mice models of EAE. Another C5aR1 inhibitor, CCX-168, has proven useful for management of anti-neutrophil cytoplasmic (ANCA) vasculitis. The soft-bodied tick *Ornithodoros moubata* complement inhibitor (OmCI) is another small molecule C5 inhibitor that has been shown to abolish complement activation in porcine and human serum (203), and could serve as a candidate molecule for CNS inhibition.

One of the fields that is gaining more traction in recent years is the development of tools to transiently open the BBB in order to achieve the selective delivery of inhibitors. These methods include the use of carrier-mediated transport mechanisms, peptidomimetics, lipidization and nanoparticles, among others. This was extensively reviewed by Kasinathan et al. (204). One of the most promising tools is the use of focused ultrasounds that achieve local and temporary disruption of the BBB confined to a target site (205–207). This method consists in the use of air entrapped in albumin nanoparticles that encapsulate the drug to be delivered. When ultrasounds are applied to the area of interest, oscillation of the bubbles in those cerebral capillaries leads to transient BBB opening allowing for the delivery of the compound of interest to the BBB. This allows for the specific delivery of therapies to the areas of active pathology all while preventing systemic effects. This method has been successfully applied in mouse models of AD where use of anti-Aβ antibodies significantly reduced plaque burden (208). Conjugating these particles to C5 antibodies such as eculizumab or other complement inhibitors could allow for the delivery of this compound to the CNS in patients with progressive MS where the inflammatory events are relatively encapsulated. It is important to note that the transient opening of the BBB to deliver these compounds also entails potential risks. Among them is the leakage of systemic factors through the BBB and into the brain parenchyma. One of these proteins is fibrinogen, which in addition to being a member of the coagulation cascade, is also involved in proinflammatory functions (209) and neurological disease (46). In EAE, fibrinogen extravasation induces microglia activation, axonal damage and active T cell recruitment (210). Fibrinogen deposition has also been described in the brains of progressive MS where high levels of this glycoprotein in the parenchyma correlated with a reduced neuronal density (211). Albumin infusion into young rodent brains has also been shown to induce astrocytic TGFβ signaling, aberrant electrocorticographic activity with increased predisposition to seizures, and cognitive impairment. Therefore, apart from the exposure of the BBB to systemic circulation, the use of albumin nanoparticles carries the additional risk of incidentally delivering this protein to the CNS where it may exert these noxious effects (111). Another big hurdle in progressive MS is the timing and dosing of these therapies. Complement proteins have a high turnover rate. This requires eculizumab to have a biweekly administration regimen in patients with AQP4+ NMOSD (212). Chronic progressive lesions in MS have a constant relentless but slow progression at the sides of the plaques. Thus, it remains to be elucidated whether targeted inhibition of complement in this disease could curb the inflammatory phenotype to an extent that chronic therapy would not be needed or whether periodic sessions would be necessary to taper chronic complement activation and prevent progression.

Another approach to the delivery of complement inhibitors in MS is the use of Trojan horse technologies. This involves the use of a fusion protein that contains the drug of interest bound to a delivery component that utilizes the specific receptors in the BBB. This has been successfully purposed for AD models where an anti-Aβ fragment was bound to an antibody fragment that recognized the insulin receptor in the BBB allowing for its bidirectional passage through the BBB (213). The transferrin receptor (TfR) a brain endothelial cell target has also been largely targeted to facilitate receptor-mediated transcytosis across the BBB (214). Again, fusing of these targeted peptides with complement inhibitors could allow for their delivery to the CNS. In order to ensure retention of these targeted antibodies in the CNS, a MMP target domain could be included in the chimeric molecule that leads to its release by enzymatic cleavage after delivery to the CNS. This would prevent the bidirectional exit of these molecules once delivered and ensure its retention in the CNS.

## Concluding remarks

In this review we have evaluated the role of complement activation in multiple sclerosis. The activation of the early complement components, C1q and C3 with generation of C3b, iC3b, C3d, may exert a double function. On the one hand, these could aid in promoting the myeloid cell-mediated phagocytosis of myelin degradation products and apoptotic bodies in the white matter. This could be a physiological process, but its aberrant activation could also explain the myelin degradation that occurs at the rim of progressive MS white matter lesions as well as the conversion of microglia to a reactive, disease-associated, phenotype. On the other hand, the activation of these components, and especially C3, in the gray matter could lead to synaptic loss through dysregulated synaptic pruning. The terminal components of the complement cascade may also be exerting a “double-edge sword” effect in MS. Initially during acute disease, MAC formation on the myelin membrane could lead to primary demyelination. Once the disease progresses towards chronicity, MAC could have a regenerative function by promoting apoptosis of inflammatory cells and degenerating oligodendrocytes. The source of these complement proteins could be systemic in the early stages by egression through a compromised BBB or from the TLT at the meninges. As disease progresses, complement proteins can also be intrinsically synthesized by intraparenchymal and perivascular myeloid cells, astrocytes, and neurons themselves. The dual function of the different components of the complement in different phases of the disease adds complexity to the development inhibitors and modulators. However, their potential role in this disease makes them a very appealing target. As an example, in animal models, local inoculation of the C3 inhibitor Crry was able to rescue synaptic loss in EAE, and the systemic use of C6 antisense oligonucleotide prevented the development of EAE. Currently the best clinical evidence for the use of complement inhibitors in neurologic disease comes from the use of C5 antibody eculizumab in AQP4+ NMOSD and MG. However, the possible beneficial role of MAC in chronic MS, the inefficacy of C5 inhibition in EAE models and the lower ability of eculizumab to penetrate a preserved BBB makes it unclear whether this antibody could have a beneficial effect in MS, and in particular progressive MS. Therefore, more resources and investigation are required for the development of new effective complement inhibitors in MS. New small molecule and antibody C3, C5, factor B and D inhibitors are currently on human clinical trials for other neurologic and non-neurologic conditions. These compounds could thus be assessed for safety and efficacy in MS patients. In addition, it is essential to develop methods that can ensure effective delivery of these inhibitors to affected areas in the CNS while avoiding its systemic side effects. One of the most intriguing approaches for MS patients with focal GM and WM lesions would be the use of focused ultrasound to selectively and transiently open the BBB at the affected areas of interest.

All these complement-based therapies and delivery methods are still on their early stages of development, but we hope that with the regained interest in the role of the complement system in neuroinflammation we will soon be able to develop effective complement-based therapeutics for MS, and especially for the progressive form of the disease for which treatment options are currently scarce.

## Author contributions

NSC and OS have contributed equally to this work. NSC and OS contributed to conception of this review. NSC wrote the first draft of the manuscript. OS contributed to manuscript revision, read, and approved the submitted version. All authors contributed to the article and approved the submitted version.

## Conflict of interest

The authors declare that the research was conducted in the absence of any commercial or financial relationships that could be construed as a potential conflict of interest.

## Publisher’s note

All claims expressed in this article are solely those of the authors and do not necessarily represent those of their affiliated organizations, or those of the publisher, the editors and the reviewers. Any product that may be evaluated in this article, or claim that may be made by its manufacturer, is not guaranteed or endorsed by the publisher.
